# *Butia *spp. Pulp: Composition, Antioxidant Properties, Biological Effects, and Food Applications

**DOI:** 10.1007/s11130-026-01571-4

**Published:** 2026-10-01

**Authors:** Chaiane Goulart Soares, David Aminagat Callirgos Romero, Marjana Radünz, Elisa dos Santos Pereira, Márcia Vizzotto, Jessica Fernanda Hoffmann, Cesar Valmor Rombaldi, Chirle Raphaelli

**Affiliations:** 1 Federal University of Pelotas (UFPel), R. Gomes Carneiro, No. 1, Balsa, Pelotas, 96010-610 Rio Grande do Sul Brazil; 2 University of Vale do Rio dos Sinos (Unisinos), Technological Institute for Food and Health (itt Nutrifor), Av. Unisinos, No. 950, Cristo Rei, São Leopoldo, 93022-750 Rio Grande do Sul Brazil

**Keywords:** Bioactive compounds, Polyphenols, Phytochemicals, Volatile compounds

## Abstract

The genus *Butia* (Arecaceae), native to southern South America, has attracted increasing scientific interest because of the nutritional composition, bioactive compounds, and technological potential of its fruits. This review critically examines the physicochemical composition, bioactive compounds, antioxidant capacity, biological effects, postharvest management, preservation strategies, and food applications of *Butia* pulp. Available evidence indicates that the pulp of different *Butia* species contains dietary fiber, minerals, phenolic compounds, carotenoids, vitamin C, and predominantly unsaturated fatty acids, although its composition varies among species, maturity stages, and growing conditions. Preclinical studies have reported antioxidant activity and possible anti-inflammatory, hypolipidemic, antidiabetic, chemopreventive, and antitumoral effects; however, these findings are mainly derived from in vitro studies and animal models, and they have not been clinically validated in humans. *Butia* pulp has also been experimentally incorporated into beverages, dairy products, frozen desserts, and dehydrated ingredients, while refrigeration, freezing, modified-atmosphere storage, drying, and freeze-drying have been investigated in different studies as strategies for maintaining postharvest quality. Nevertheless, knowledge remains limited for many Butia species, and studies addressing postharvest deterioration, volatile compounds, bioaccessibility and bioavailability, sensory acceptance, analytical standardization, and industrial-scale food applications are still scarce. Future research should expand investigations to underexplored species, validate preservation and processing strategies under conditions approaching industrial scale, and evaluate emerging non-destructive and non-thermal approaches to improve quality control and extend shelf life.

**Supplementary Information:**

Uma versão online contém material suplementar disponível em https://doi.org/10.1007/s11130-026-01571-4.

## Introduction

Underutilized native fruits have attracted growing scientific interest because of their nutritional composition, bioactive compounds, and potential contribution to dietary diversification and sustainable food systems [1]. However, only 22.5% of Brazilian adults consume the fruit and vegetable intake recommended by the World Health Organization, corresponding to at least 400 g per day or five daily servings [2]. In this context, *Butia*, a genus of the Arecaceae family native to southern South America, has gained attention as a source of nutrients and bioactive compounds with potential food applications [3, 4].

**Fig. 1:**
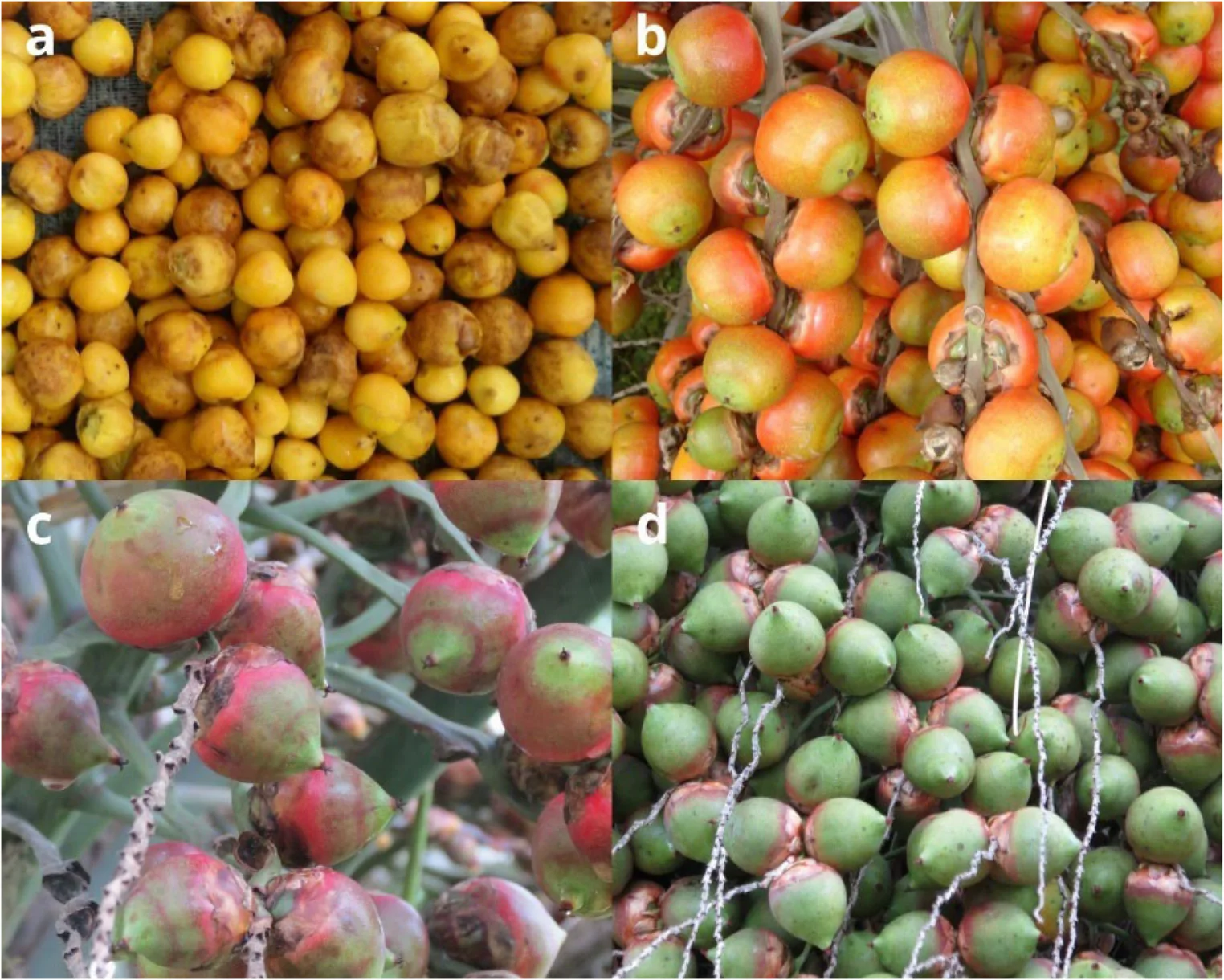
Morphological diversity of Butia spp. (a) B. catarinensis (photograph by Marcelo Piske Eslabão); (b) B. odorata; (c) B. paraguayensis; and (d) B. yatay (photographs by Gustavo Heiden)

The genus *Butia* comprises approximately 20–25 subtropical species distributed mainly in Brazil, Uruguay, Paraguay, and Argentina, with broad occurrence in southern, southeastern, and midwestern Brazil [5]. The morphological diversity of fruits among representative species of the genus is illustrated in Fig. 1. Species such as *Butia odorata*, *Butia eriospatha*, and *Butia catarinensis* produce juicy, aromatic fruits with fibrous, slightly acidic pulp suitable for fresh consumption and incorporation into food products [6]. These fruits are also traditionally consumed and processed by local communities, contributing to the valorization of regional food resources and production chains [6, 7].

*Butia* pulp contains nutrients and phytochemicals of potential nutritional and functional relevance, including dietary fiber, minerals, vitamin C, carotenoids, and phenolic compounds, such as catechin, epicatechin, and rutin [8, 9]. These constituents contribute to its antioxidant properties and have stimulated research into its biological potential. Preclinical studies have reported antioxidant, anti-inflammatory, hypolipidemic, antidiabetic, chemopreventive, and antitumor effects associated with *Butia* pulp and extracts, including enzyme inhibition, protection against oxidative and genotoxic damage, reduced viability of tumor cells, and decreased formation of preneoplastic lesions in animal models [3, 10–15].

The broader utilization of *Butia* fruits is constrained by their high perishability and limited postharvest stability. Some species, including *B. eriospatha*, have been described as non-climacteric, whereas in *B. capitata*, ripening is closely associated with fruit abscission and rapid quality loss at room temperature [16, 17]. High moisture content, ranging from approximately 78 to 90%, together with the susceptibility of bioactive compounds to oxidative degradation, further limits storage and large-scale commercialization [18]. Consequently, *Butia* pulp has been incorporated into juices, fermented beverages, dairy products, jams, frozen desserts, and other regional foods as a strategy to expand its utilization and valorize its sensory, nutritional, and bioactive attributes [19–21].

Although previous reviews have addressed the chemical composition and general uses of the genus *Butia* [22, 23], evidence specifically addressing the edible pulp remains fragmented.

Previous syntheses have frequently combined evidence from pulp, seeds, oils, leaves, and by-products, limiting the independent assessment of the edible fraction. Moreover, differences in species, geographical origin, maturity stage, analytical methodology, and data expression hinder direct comparisons and an integrated interpretation of the relationships among composition, biological activity, processing, and food applications.

Therefore, this review addresses the following research questions: (i) what are the physicochemical characteristics and bioactive compound profiles of *Butia* spp. pulp? (ii) how are these characteristics related to its antioxidant properties, health-related biological effects, postharvest behavior, processing, and food applications? and (iii) what methodological and knowledge gaps currently limit the interpretation and practical application of these findings? Accordingly, this review critically integrates the available compositional, biological, and technological evidence, with particular attention to the research gaps limiting the broader utilization of *Butia* pulp.

## Search Strategy and Study Selection

A structured literature search was conducted to enhance transparency and reproducibility of this narrative critical review, following relevant PRISMA reporting recommendations [24] where applicable, as described in Supplementary Material S1.

## Composition and Physicochemical Properties of *Butia* spp. pulp

### Physicochemical Properties and Pulp Yield

Fruits of the genus *Butia* show substantial variation in morphological and biometric traits and pulp yield among species, genotypes, and growing regions, reflecting genetic and environmental influences on fruit characteristics [4, 25]. The fruits are generally ovoid to globose, measure approximately 3–4 cm in diameter, and may reach pulp yields above 80% in some genotypes, an advantageous characteristic for food processing [6, 9, 18].

As summarized in Table 1, *Butia* pulp is generally characterized by low pH, relatively high titratable acidity (TTA), and variable soluble solids (SS content). During ripening, the reduction of organic acids and the accumulation of sugars increase the SS/TTA ratio and perceived sweetness, although the magnitude and direction of these changes may vary among species and genotypes [4]. Fruits with higher ratios may therefore be more acceptable for fresh consumption, whereas greater acidity may be advantageous for selected processed products, including jams and beverages [31]. Fruit color ranges from yellow to intense red and represents an important quality attribute that may reflect differences in carotenoid composition and concentration [9]. However, physicochemical values vary with species, genotype, ripening stage, growing conditions, and postharvest handling.

**Table 1:** Physicochemical characteristics and proximate composition of *Butia* pulp reported in the literature

Espécie	pH	TTA (%)	SS (°Brix)	Umidade (%)	Proteína	CHO	Lipídios	Cinza	Fibra	Base	Referência
*B. capitata*	NR	NR	NR	84,45	NR	NR	17,94	NR	NR	DB	[26]
*B. capitata*	NR	NR	NR	82,34	4.19	70,84	19,37	3.28	NR	DB	[6]
*B. catarinensis*	3.33	1.28	12,88	82,83	5.01	51,53	9,13	3.74	30,59a	DB	[27]
*B. catarinensis*	2.80	3.22	22.08	NR	NR	NR	NR	NR	NR	WB	[28]
*B. eriospatha*	2,94	1.90	9,53	87,18	5,47	64,20	1.55	4.64	23,11a	DB	[27]
*B. odorata*	3.50	1,60	12.39	81,22	3.74	14,50	6,76	3,30b	8,99b	DB	[18]
*B. odorata*	3.00	1.21	13,86	NR	NR	NR	NR	0.54	2,18a	WB	[9]
*B. odorata*	3.50	1.99	12,92	85,80	5.70	NR	NR	4,79	26,76	DB	[29]
*B. odorata*	2.99	1,79	12,97	82,20	4,27	NR	NR	3.93	21,07	DB	[29]
*B. odorata*	3.53	1.69	11,48	84,23	4.44	NR	NR	4.06	23.15	DB	[29]
*B. odorata*	3,20	1,50	11,83	83,29	4.41	NR	3.49	3.49	8,79b	DB	[30]
*B. Yatay*	NR	NR	NR	80.00	6.50	57,00	4.50	3.00	28,50	DB	[25]

Proximate composition values are expressed on a dry basis (DB) whenever sufficient sample-specific moisture or dry matter information was available in the original study

Values for which reliable conversion was not possible are retained on a wet basis (WB) and identified accordingly

For studies reporting individual samples, values were converted individually to DB before calculating the corresponding means

Moisture is reported as described in the original studies

pH is dimensionless, total titratable acidity (TTA) is expressed as % citric acid, and soluble solids (SS) as °Brix

*CHO* carbohydrates, *NR* not reported, *DB* dry basis, *WB* wet basis, *TDF* total dietary fiber, *CF* crude fiber., *Ref* reference

ᵃTDF; ᵇCF

### Proximate Composition and Dietary Fiber

The proximate composition of *Butia* pulp is characterized by high moisture content, ranging from 80 to 92% on a fresh-weight basis (Table 1). This contributes to the susceptibility of the pulp to microbial and biochemical deterioration and partly explains its limited postharvest stability [31, 32].

Protein and lipid contents are generally low on a fresh-weight basis, and reported energy values range from 59.00 to 83.34 kcal/100 g of fresh pulp [6, 25] Total amino acids determined after protein hydrolysis ranged from 0.91 to 2.89% (w/w), with *B. lallemantii* showing the highest concentrations of total and essential amino acids among the species evaluated, including His, Thr, Met, Val, Phe, Leu/Ile, and Lys [33]. The lipid fraction is predominantly composed of unsaturated fatty acids. Although dietary lipids can influence carotenoid solubilization and micellization, the relatively low lipid content of *Butia* pulp may limit this process, and the extent to which the endogenous lipid fraction contributes to carotenoid bioaccessibility remains insufficiently characterized [26, 33]. Ash contents are also relatively low, although direct comparisons remain dependent on the analytical and expression bases used in each study [12, 18].

Carbohydrates constitute a major fraction of the pulp and may include soluble sugars, pectic substances, and other structural polysaccharides that contribute to sweetness, texture, viscosity, and processing behavior. However, reported carbohydrate values are not always directly comparable because some studies determined specific fractions, whereas others estimated total carbohydrates by difference [8, 18]. This methodological heterogeneity should be considered when interpreting apparent differences among species.

Reported fiber contents also vary widely among species and studies. Direct comparison is limited because the available literature includes both total dietary fiber and crude fiber values expressed on fresh- or dry-weight bases (Table 1). Nevertheless, the results consistently indicate that *Butia* pulp contains a relevant fibrous fraction that may contribute to dietary fiber intake and influence the texture and processing behavior of derived products [9, 18, 25, 34].

### Mineral Composition

*Butia* pulp is characterized by potassium as the predominant mineral, followed by calcium and magnesium, together with lower concentrations of iron, zinc, copper, and other trace elements [6, 27, 28, 35]. Potassium concentrations of approximately 430–460 mg/100 g of fresh pulp have been reported, indicating that the fruit may contribute to dietary potassium intake [27].

A comprehensive characterization of the mineral composition of the edible portions of *B. catarinensis* and *B. eriospatha* was provided [27], including calcium, potassium, sodium, magnesium, iron, zinc, manganese, copper, phosphorus, and selenium. Similarly, potassium was identified as the predominant mineral [29], with concentrations ranging from 1183 to 1758 mg/100 g on a dry-weight basis, followed by magnesium (47–79 mg/100 g) and calcium (42.0–54.0 mg/100 g). As these values were expressed on a dry-weight basis, they are not directly comparable with those reported for fresh pulp. Magnesium concentrations also varied among harvests, particularly in (Table 1) *B. eriospatha*, suggesting that growing and harvest conditions may influence mineral accumulation. Selenium was below the detection limit in the analyzed samples [27, 29].

### Lipid Profile

*Butia* pulp generally contains less than 3% lipids on a fresh-weight basis [6]. Despite this low lipid content, its fatty acid profile is characterized by a predominance of unsaturated fatty acids, as summarized in Table S1. These account for approximately 68.6% of the fatty acids reported for *B. yatay* and about 63% for *B. capitata*, although direct comparisons should consider possible differences in analytical methodologies and expression bases among studies [25, 26].

Oleic acid is generally the predominant fatty acid in the pulp, followed by linoleic and palmitic acids [18, 26]. Lower proportions of α-linolenic acid have also been reported, and differences in the relative abundance of n-6 and n-3 fatty acids have been observed among *B. odorata* samples [18]. Although the total lipid content of the pulp is low, the predominance of unsaturated fatty acids represents a favorable nutritional characteristic of *Butia* spp. [30, 36, 37].

### Volatile Compounds

Volatile compounds are key determinants of the sensory profile of *Butia* fruits and play an important role in the aroma quality of both fresh fruit and processed products, including juices, nectars, fermented beverages, and culinary preparations [18, 26].

Among the limited studies available, esters are the predominant class of volatile compounds identified in *Butia* spp., particularly ethyl esters derived from short- and medium-chain fatty acids, which are responsible for the characteristic fruity and sweet aroma. Among the most frequently reported compounds are ethyl butanoate, ethyl hexanoate, and ethyl octanoate, widely distributed in species such as *B. odorata* and *B. capitata* [29].

In addition to esters, the volatile profile includes aldehydes, alcohols, volatile acids, and terpenes, which increase aroma complexity and contribute to sensory differences among species. Hexanal, 1-hexanol, limonene, and β-myrcene are among the representative compounds reported for the genus [31, 38].

Qualitative and quantitative differences in the composition of esters, aldehydes, alcohols, and terpenes account for much of the aromatic variability observed among *Butia* species and genotypes. These compounds are also technologically relevant because processing and storage conditions may alter their composition, thereby affecting the aroma quality and sensory properties of processed products. However, volatile profiles have been characterized in only two studies [31, 38], indicating the need for further research across additional species, genotypes, ripening stages, and processing conditions to provide a more comprehensive understanding of the sensory diversity of *Butia* fruits.

## Bioactive Compounds in *Butia* spp. Pulp

Several studies have quantified the main groups of bioactive compounds present in the pulp of different *Butia* species, and the reported values are summarized in Table S2. Despite the biological and methodological variability among studies, the available evidence indicates that *Butia* spp. pulp contains phenolic compounds, carotenoids, and vitamins of potential nutritional and functional relevance. The following sections discuss their composition, interspecific variability, and potential contribution to the nutritional and functional properties of these fruits.

### Phenolic Compounds

Total phenolic contents ranging from approximately 173.5 to 493.6 mg gallic acid equivalents (GAE)/100 g fresh weight have been reported for *Butia* spp. pulp, indicating that these fruits are a relevant source of phenolic compounds [6, 8, 9]. Beyond total content, metabolomic and chromatographic studies have revealed a complex and species-dependent phenolic composition, with marked differences among members of the genus [4].

The phenolic profile includes hydroxycinnamic and hydroxybenzoic acids, particularly chlorogenic, gallic, caffeic, ferulic, *p*-coumaric, vanillic, syringic, and ellagic acids, together with flavonoids such as catechin, epicatechin, rutin, quercetin, kaempferol, luteolin, apigenin, naringenin, and trans-resveratrol [4, 10, 33]. Chlorogenic acid has frequently been reported as the predominant phenolic compound in species such as *B. odorata* and *B. yatay* [4, 25].

Recent metabolomic studies have further demonstrated distinctive phenolic signatures among species. *B. odorata* is particularly rich in catechin, epicatechin, and rutin, whereas *B. catarinensis* shows higher concentrations of hydroxybenzoic acid derivatives, and *B. eriospatha* is characterized by a predominance of flavan-3-ols, especially epicatechin gallate [10, 36, 39]. These interspecific differences indicate that phenolic composition is an important source of chemical variability within the genus, with potential implications for the antioxidant properties of the fruits.

Phenolic acids and flavonoids identified in *Butia*, particularly catechin, epicatechin, and hydroxycinnamic acid derivatives, are widely recognized for their antioxidant properties and have been identified as important contributors to the radical-scavenging activity reported for these fruits [33, 36, 40]. They may also contribute to sensory attributes such as color, astringency, and flavor complexity [4, 40].

Although direct comparisons are limited by differences in species, genotype, ripening stage, extraction procedures, analytical methodologies, and expression bases, the total phenolic contents reported for *Butia* pulp are comparable to or higher than those of widely consumed fruits such as apple, pear, and black grape [41].

### Carotenoids

Carotenoids are important bioactive constituents of *Butia* pulp and contribute to the characteristic yellow-to-orange coloration of the fruits (Table S2). Reported total carotenoid contents range from approximately 0.38 to 4.08 mg/100 g fresh weight, depending on species, ripening stage, and analytical methodology [28, 30, 42]. The carotenoid profile includes β-carotene, β-cryptoxanthin, zeaxanthin, α-carotene, lutein, lycopene, rubixanthin, and pro-γ-carotene [9, 43].

β-Carotene, α-carotene, and β-cryptoxanthin possess provitamin A activity, thereby contributing to the nutritional relevance of *Butia* fruits [36]. Other carotenoids identified in the pulp, including lutein, zeaxanthin, and lycopene, are widely recognized for their roles in antioxidant defense and ocular health [40, 44]. However, these physiological effects have been established mainly from studies involving isolated compounds or other food matrices, and their relevance in *Butia* depends on carotenoid content, bioaccessibility, and interactions within the food matrix.

Carotenoids also contribute to the color and potential functional value of *Butia*-derived products. Nevertheless, their susceptibility to thermal, oxidative, and photoinduced degradation may reduce retention during processing and storage [4, 21, 28, 32]. Drying, thermal treatment, oxygen exposure, and prolonged storage therefore require careful control to preserve carotenoid content and product quality.

### Vitamin E

Vitamin E compounds, including tocopherols and tocotrienols, are among the lipophilic bioactive constituents identified in *Butia* spp. pulp and contribute to antioxidant protection in lipid environments [14]. In *B. capitata* pulp, total vitamin E content has been reported at approximately 0.12 mg/100 g fresh weight, with α-tocopherol identified as the predominant homolog [6, 14] (Table S2). In addition, five vitamin E homologs have been detected, indicating a certain degree of compositional diversity within this class of compounds.

Reported vitamin E contents may be influenced by genotype, fruit maturity, environmental and storage conditions, as well as methodological differences among studies [6]. Although vitamin E compounds may contribute to the antioxidant properties of the lipid fraction, the available evidence for *Butia* remains limited. Further studies involving additional species and genotypes are needed to better characterize vitamin E composition and to evaluate its stability, bioaccessibility, and retention during processing [6, 14].

### Vitamin C

Vitamin C (*L*-ascorbic acid) is an essential nutrient involved in immune function, collagen synthesis, and the absorption of non-heme iron [45]. Fruits of the genus *Butia* are recognized as important sources of vitamin C, although concentrations vary among species, genotypes, and cultivation conditions [41]. In *B. odorata* pulp, vitamin C contents ranging from 34.63 to 63.84 mg/100 g fresh weight have been reported [31, 46], whereas *B. capitata* exhibits concentrations between 43.0 and 53.6 mg/100 g fresh weight [41]. This variability may also be associated with fruit maturity, postharvest conditions, and the fruit fraction analyzed.

Compared with other native Brazilian fruits, *Butia* spp. exhibits vitamin C contents similar to those reported for araticum-do-mato (approximately 32 mg/100 g fresh weight) and higher than those observed in mandacaru-de-três-quinas (approximately 25 mg/100 g fresh weight) [19]. Although lower than the exceptionally high concentrations reported for fruits such as camu-camu, the values fall within the range reported for widely consumed fruits such as orange [42, 46].

In addition to its nutritional importance, vitamin C contributes to the antioxidant capacity of *Butia* pulp. However, its susceptibility to oxygen, heat, and storage conditions may reduce retention during processing of *Butia*-derived products [45]. Therefore, appropriate processing and preservation strategies are important to minimize vitamin C losses.

## Antioxidant Capacity and Health-Related Biological Effects

### Antioxidant Capacity

The bioactive composition of *Butia* spp. pulp provides the chemical basis for its antioxidant capacity, mainly through the combined contribution of phenolic compounds, vitamin C, carotenoids, and vitamin E [9]. Owing to their different polarities and mechanisms of action, hydrophilic and lipophilic antioxidants may act complementarily in the neutralization of reactive oxygen species [29, 40].

The antioxidant capacity of *Butia* pulp has been evaluated using several chemical assays including 2,2-diphenyl-1-picrylhydrazyl (DPPH), 2,2′-azinobis(3-ethylbenzothiazoline-6-sulfonic acid) (ABTS), ferric reducing antioxidant power (FRAP), and oxygen radical absorbance capacity (ORAC), as summarized in Table S3. These methods assess different antioxidant mechanisms, mainly through electron transfer or hydrogen atom transfer reactions [42, 47]. The available studies consistently demonstrate radical-scavenging capacity and reducing power in *Butia* pulp, although the magnitude of the response varies according to species, genotype, collection site, ripening stage, and analytical conditions. Therefore, the reported values should be interpreted as complementary indicators of antioxidant potential rather than as a strict ranking among species. Moreover, chemical assays do not reproduce the complexity of biological systems and cannot be used to directly infer physiological effects.

### Bioaccessibility and Bioavailability of Bioactive Compounds

The biological effects associated with *Butia* spp. pulp depend not only on the initial concentration of its bioactive compounds but also on their release from the food matrix during digestion. Bioaccessibility refers to the fraction of a compound released during gastrointestinal digestion and made available for intestinal absorption, whereas bioavailability also encompasses the subsequent processes of absorption, metabolism, distribution, and excretion [48–52]. Because studies on *Butia* have relied mainly on simulated gastrointestinal digestion models, the available evidence relates primarily to bioaccessibility rather than to complete in vivo bioavailability.

Simulated digestion studies indicate that gastrointestinal conditions may alter both the concentration of bioactive compounds and the antioxidant activity of the pulp. DPPH radical inhibition values ranging from 79.57 to 88.12% were reported after the intestinal phase in *B. odorata* extracts [8, 53], depending on the geographical origin of the fruits. Although antioxidant activity remained high after digestion, the changes observed throughout the process indicate that gastrointestinal conditions may affect the stability and reactivity of antioxidant compounds. Similarly, a decrease in total phenolic content has been reported after simulated digestion, suggesting that only part of the initially quantified fraction remains detectable and potentially available for absorption [53].

The behavior of individual compounds during digestion is not uniform. Some phenolic compounds, such as catechin, appear to be more stable than the total phenolic fraction, indicating structural differences in their resistance to pH changes, enzymatic activity, and interactions with the food matrix [8, 12]. Therefore, a reduction in total phenolic content does not necessarily imply an equivalent loss of all individual constituents.

Other groups of bioactive compounds follow different bioaccessibility mechanisms. For carotenoids, release from the food matrix and incorporation into mixed micelles during the intestinal phase are key steps for absorption, whereas vitamin C is particularly susceptible to oxidation and degradation [4, 54]. However, specific information on the digestive stability, micellization, and bioaccessibility of these compounds in *Butia* pulp remains limited.

A fraction of the phenolic compounds may be associated with dietary fiber and structural polysaccharides in the pulp, thereby restricting their release in the upper gastrointestinal tract [36]. These compounds may reach the colon and be transformed by the gut microbiota into lower-molecular-weight metabolites [15, 53]. After absorption, phenolic compounds commonly undergo phase II reactions, including glucuronidation, sulfation, and methylation, generating derivatives that may differ from the molecules originally present in the fruit [55, 56]. However, these metabolic pathways have not yet been specifically characterized for *Butia*-derived compounds, and their physiological contribution therefore remains insufficiently understood.

Standardized digestion models, such as INFOGEST, have improved the reproducibility and comparability of this type of research. Nevertheless, they do not fully reproduce intestinal absorption, colonic fermentation, hepatic metabolism, or interindividual variability [12, 57]. Future studies should therefore integrate standardized digestion models, intestinal transport systems, colonic fermentation studies, metabolomic approaches, and human trials. Such integration would allow a more accurate characterization of the metabolic fate of *Butia* bioactive compounds and clarify the extent to which bioaccessibility translates into bioavailability and physiological effects.

### Health-Related Biological Effects

As summarized in Table 2, preclinical studies indicate that *Butia* spp. pulp and extracts have shown antioxidant and anti-inflammatory activity and have suggested hypolipidemic, antidiabetic, antitumor, and chemopreventive potential, depending on the experimental model employed [3, 11, 58]. The available evidence derives mainly from biochemical assays, cell-based models, and animal studies. To date, no clinical trials have confirmed these effects in humans [8, 11, 12].

**Table 2:** Experimental evidence of biological effects and functional potential associated with *Butia* spp. pulp

Species	Sample and extractionapproach	Experimental model	Main biological findings	Potential biological relevance	Main limitations	Reference
*B. capitata*	Oven-dried pulp; hydroalcoholic extract (60:40 methanol: water)	In vitro – human colorectal cancer cells (HT-29)	Dose- and time-dependent inhibition of tumor cell growth	Antitumoral and functional/nutritional relevance	Evidence restricted to an in vitro model; interactions among identified compounds remain unclear	[13]
*B. capitata*	Aqueous pulp extract (50:50 pulp: water), orally administered	In vivo – Wistar rats with induced colorectal carcinogenesis	Reduction of aberrant crypt foci, lower IL-1 expression, and preservation of colon tissue	Chemopreventive and anti-inflammatory	Animal model only; mechanisms not fully elucidated	[14]
*B. eriospatha*	Lyophilized pulp; acidified methanolic extract	In vitro – Caco-2 cells	Reduction oxidative stress and protection against DNA damage	Antioxidant activity and cytoprotective	No in vivo and clinical validation	[49]
*B. odorata*	Edible portions (pulp and peel); ethanolic extract	In vitro – antioxidant and enzyme assays	α-Glucosidase inhibition; scavenging of DPPH•, OH•, and NO• radicals	Antioxidant and antidiabetic potential	Absence of in vivo validation; reduced stability after intestinal digestion	[10, 50]
*B. odorata*	Frozen pulp; hydroethanolic extract (70:30 ethanol: water)	In vitro – glioma cell lines (C6 and U87MG)	Reduced cell viability, proliferation, and colony formation; decreased ROS and nitrite production	Antitumoral (glioma model), antioxidant and anti-inflammatory	Lack of in vivo studies; limited molecular evaluation in human cell lines	[3]
*B. odorata*	Frozen pulp and peel; ethanolic extract	In vitro – cervical cancer cell lines (SiHa and C33a)	Significant reduction in tumor cell viability	Antitumoral (cervical model) and antioxidant	Limited response in other cell lines; evidence restricted to cellular models	[10]
*B. odorata*	Lyophilized hydroalcoholic pulp extract, orally administered	In vivo – rats with induced hyperlipidemia	Reduced triglycerides, total cholesterol, and LDL; increased HDL and PON1 activity; lower inflammatory markers	Hypolipidemic, cardiometabolic, and anti-inflammatory effects	Acute model and short treatment duration; absence of clinical studies	[11]
B. odorata, B. catarinensis, B. eriospatha	Lyophilized pulp; hydroethanolic extract (70:30 ethanol: water)	In vitro – simulated gastrointestinal digestion	Inhibition of α-glucosidase and α-amylase; antioxidant capacity maintained after digestion, although reduced after the intestinal phase	Antidiabetic and antioxidant	Bioactivity depends on digestive stability; bioaccessibility does not confirm bioavailability	[12]

Biological effects were assessed using heterogeneous sample preparation procedures, extraction methods, experimental models, doses, and exposure conditions

Therefore, direct comparisons between studies should be interpreted cautiously, as these methodological factors may significantly influence the magnitude and interpretation of the reported biological responses

The available evidence should be considered primarily as preclinical evidence supporting further investigation rather than as confirmation of clinical benefits in humans

*IL-1* interleukin-1, *DPPH*• 2,2-diphenyl-1-picrylhydrazyl radical, *OH*• hydroxyl radical, *NO*• nitric oxide radical, *LDL* low-density lipoprotein, *HDL* high-density lipoprotein, *PON1* paraoxonase 1, *ROS* reactive oxygen species, *HT-29* human colorectal adenocarcinoma cell line, *Caco-2* human colorectal adenocarcinoma cell line, *C6* rat glioma cell line, *U87MG* human glioblastoma cell line, *SiHa* human cervical squamous cell carcinoma cell line SiHa, *C33A* human cervical carcinoma cell line C33A

In in vitro models, *Butia* extracts have shown the ability to neutralize reactive oxygen and nitrogen species and to inhibit enzymes involved in carbohydrate metabolism. In particular, inhibition of α-glucosidase and α-amylase has been reported after simulated digestion of extracts from different species, providing preliminary mechanistic evidence supporting their antidiabetic potential through the possible modulation of carbohydrate digestion [10, 12, 53]. These activities have been associated mainly with catechins, rutin, phenolic acids, and other phenolic compounds present in the pulp. Nevertheless, enzyme inhibition represents preliminary mechanistic evidence and requires validation in clinical models.

Cell-based studies have expanded this evidence by demonstrating effects in biological systems. In Caco-2 intestinal cells, *Butia* polyphenols reduced intracellular oxidative stress and protected against DNA damage, supporting their antioxidant and cytoprotective activity [49]. Likewise, *B. odorata* extracts reduced the production of reactive oxygen species and nitrites and modulated inflammation-related markers in glioma cell lines [3].

Antitumor potential has been evaluated in different cellular models. In human HT-29 colorectal cancer cells, hydroalcoholic extracts of *B. capitata* inhibited tumor cell growth in a dose- and time-dependent manner [13]. In C6 and U87MG glioma cell lines, *B. odorata* extract reduced viability, proliferation, and colony formation [3]. Similarly, reduced viability was observed in the cervical cancer cell lines SiHa and C33a [10]. These findings support antitumor activity under controlled experimental conditions, although the magnitude of the response depends on extract composition, cell line, exposure time, and concentration.

Animal studies provide additional evidence of possible systemic effects. Oral administration of *B. odorata* extract to rats with induced hyperlipidemia reduced serum triglycerides, total cholesterol, and LDL cholesterol, while increasing HDL cholesterol and paraoxonase 1 activity and decreasing inflammatory markers [10]. These findings provide preliminary evidence of hypolipidemic and anti-inflammatory effects in the evaluated experimental model.

Chemopreventive effects have also been reported in experimental models of colorectal carcinogenesis. Administration of an aqueous *B. capitata* pulp extract was associated with reduced formation of aberrant crypt foci (early abnormal lesions of the colonic crypts), decreased IL-1 expression, and preservation of colonic tissue structure [14]. These findings suggest possible chemopreventive and anti-inflammatory effects during the early stages of carcinogenesis. Recent evidence of protection against colitis-associated colorectal tumorigenesis further strengthens interest in this species, although the underlying mechanisms require further clarification.

Interpretation of these findings should consider the heterogeneity among studies in terms of species, ripening stage, sample preparation, extraction method, chemical composition, dose, treatment duration, and biological model. In addition, some concentrations used in cell culture studies may exceed those attainable through dietary intake of the fruit, thereby limiting direct extrapolation to human physiology. Although animal models allow the assessment of systemic responses, they do not ensure that comparable effects will occur after habitual consumption of *Butia*-based foods.

## Postharvest Management and Storage of *Butia* spp. Fruits

### Harvest Maturity and Initial Postharvest Practices

Determining the optimal harvest stage represents one of the main challenges for the postharvest conservation of *Butia* fruits because of the high genetic and phenotypic variability among species and accessions, together with the lack of standardized maturity indices. Fruits harvested at advanced ripening stages generally exhibit higher concentrations of soluble solids and bioactive compounds, as well as greater aromatic intensity. However, they are also more susceptible to mechanical damage and deterioration, which reduces shelf life and limits commercial utilization [42, 44].

Initial postharvest practices generally include washing followed by sanitization through immersion in sodium hypochlorite solutions to reduce the surface microbial load and minimize storage losses [17, 21]. Despite the widespread use of these procedures, little information is available on the microbiological evolution of *Butia* fruits during storage, including fungal incidence and the microorganisms primarily responsible for spoilage. Addressing these gaps would improve the understanding of the biological factors underlying postharvest losses and support the development of more effective preservation strategies.

### Storage Conditions and Quality Preservation

Storage temperature and packaging systems directly influence postharvest quality. Storage at 5 °C was compared in minimally processed *B. catarinensis* fruits using open trays, multilayer plastic packaging, and modified-atmosphere packaging containing 2% O_2_, 5% CO_2_, and 93% N_2_ [28]. Fruits stored in open trays were no longer suitable for consumption after 15 days, whereas packaged fruits maintained acceptable quality for up to 25 days under refrigeration. Packaging also contributed to preserving carotenoids, antioxidant capacity, and ascorbic acid, although it promoted greater fruit softening, highlighting a trade-off between bioactive compound retention and texture preservation during storage.

In *B. capitata*, storage at 25–27 °C accelerated postharvest ripening, increasing pH and total soluble solids while reducing titratable acidity and pulp firmness [17, 59]. Although these parameters are useful indicators of quality evolution, differences among species, maturity stages, packaging conditions, and experimental designs still preclude the establishment of general recommendations regarding storage temperature and shelf life.

Although *Butia* fruits are generally considered non-climacteric, the available evidence regarding their respiratory behavior is not entirely consistent. One study reported increased respiratory activity during the postharvest ripening of immature *B. capitata* fruits [59], whereas another study observed differences in respiratory metabolism among palm species. This variability may be associated with species, maturity stage, storage conditions, or mechanical stress during handling [17], although these relationships require further experimental confirmation [28].

### Non-Destructive Approaches for Maturity and Quality Assessment

Non-destructive approaches could help address the lack of objective criteria for determining maturity and monitoring the postharvest quality of *Butia* fruits. Near-infrared spectroscopy, hyperspectral imaging, and machine vision systems have shown potential for fruit classification, internal quality assessment, and early defect detection in other fruit matrices [60–62]. However, their application to *Butia* remains limited. Future studies should validate their performance against physicochemical and sensory attributes and assess their technical and economic feasibility under commercial conditions.

## Processing and Preservation of *Butia* spp. pulp

### Pulp Extraction and Stabilization

Pulp extraction represents the first stage in the processing of *Butia* fruits and directly influences processing yield and final product quality. At industrial or pilot scale, mechanical pulpers or extractors equipped with metallic sieves of different mesh sizes are commonly used to separate the edible fraction from the endocarp, seeds, and part of the fibrous material [63]. At artisanal or laboratory scale, pulp extraction is generally performed manually or by mechanical blending followed by filtration [34]. Processing conditions, particularly sieve mesh size and extraction intensity, may influence fiber retention, pulp consistency, and extraction yield, and should therefore be considered when comparing studies and defining processing specifications for different products.

Once extracted, the pulp has a high moisture content and contains oxidative enzymes, including polyphenol oxidase and peroxidase, which promote microbial growth, enzymatic browning, and the degradation of phenolic compounds during storage [64]. Thermal treatments, including blanching and pasteurization, are commonly applied to reduce microbial load and inactivate these enzymes, thereby improving product stability and extending shelf life [65, 66]. However, excessive heat may accelerate the degradation of vitamin C and other thermolabile compounds while adversely affecting color, aroma, and sensory quality [32, 63]. The available evidence remains insufficient to define processing conditions capable of simultaneously ensuring microbiological safety, enzymatic inactivation, and the preservation of nutritional and sensory quality.

### Conventional Preservation Methods

Freezing at − 18–− 20 °C remains the most widely used strategy for long-term pulp preservation. Although it effectively maintains microbiological stability, oxidative reactions may continue, albeit at a reduced rate, during storage. In *B. odorata* pulp stored at − 18 °C for 12 months, reductions in phenolic compounds, flavonoids, and vitamin C were reported, whereas some carotenoids exhibited greater stability [20]. Besides storage temperature, oxygen exposure, light, temperature fluctuations, and packaging permeability may also influence the retention of bioactive compounds. In this context, vacuum packaging or oxygen-barrier materials could help minimize oxidative losses, although their effectiveness has not yet been specifically evaluated for *Butia* pulp.

Dehydration reduces water availability and enables the production of powders and shelf-stable ingredients, although the retention of bioactive compounds depends strongly on the drying technology employed. Using foam-mat drying at 60–80 °C with albumin and xanthan gum, recent research reported vitamin C losses ranging from 77.96 to 92.68% relative to fresh pulp, under the specific drying conditions evaluated, decreasing from 56.03 mg/100 g to 4.10–12.35 mg/100 g after drying [32]. These losses mainly result from the combined effects of heat, oxygen exposure, and processing time, while the degradation of phenolic compounds and carotenoids may also contribute to browning and changes in color and acidity of the dehydrated product.

Freeze-drying, in contrast, removes water under low-temperature and reduced-pressure conditions, generally providing greater retention of phenolic compounds, carotenoids, and antioxidant capacity [41, 67]. Apparent increases in the concentration of some carotenoids have also been reported after processing, probably reflecting enhanced extractability resulting from cell matrix disruption or structural changes rather than a true increase in carotenoid content [63]. These analytical increases should therefore be interpreted together with changes in antioxidant activity and other oxidation-sensitive constituents. Collectively, the available evidence suggests that freeze-drying preserves the functional quality of *Butia* pulp more effectively than conventional hot-air drying, although its higher operational cost and energy demand may limit large-scale industrial application.

### Food Applications of *Butia* spp. pulp

**Fig. 2:**
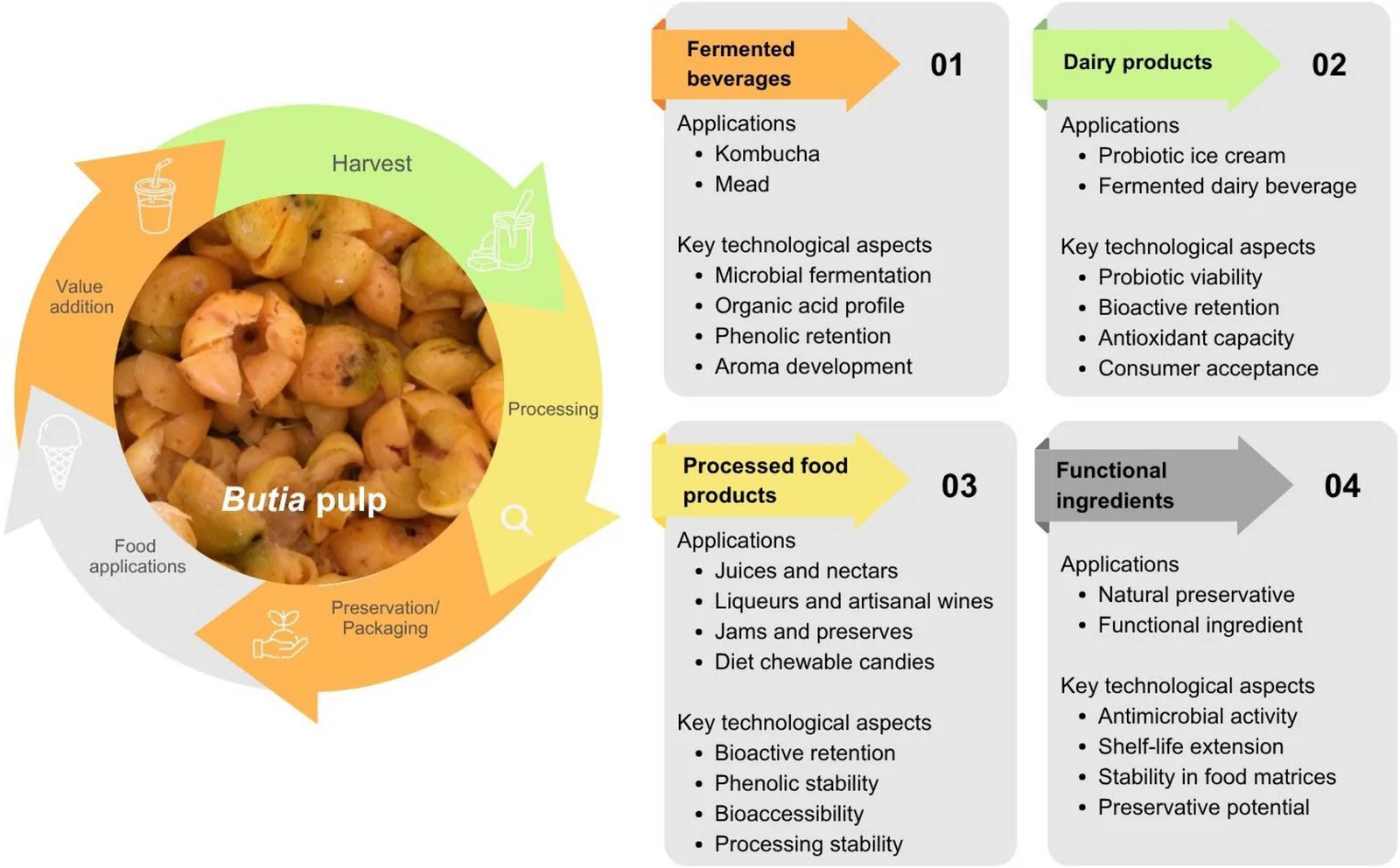
Integrated processing chain of *Butia* pulp, highlighting the main food application categories discussed in this review and their associated technological aspects

The distinctive sensory attributes and bioactive-rich composition of *Butia* spp. pulp have supported its incorporation into an increasing variety of food products, ranging from fermented beverages and dairy products to confectionery and functional ingredients [21, 63]. A schematic overview of the processing chain of *Butia* spp. pulp, highlighting representative applications and the main processes involved in each product category, is presented in Fig. 2.

### Fermented Beverages

*Butia* spp. pulp has shown potential both as a fermentation substrate and as a source of sensory differentiation. In kombucha-type beverages, *B. eriospatha* pulp favored the establishment of a microbial community dominated by acetic acid bacteria, particularly *Gluconobacter* and *Komagataeibacter*, promoting the production of gluconic and glucuronic acids while limiting ethanol accumulation [68]. Total phenolic content remained relatively stable during fermentation, indicating satisfactory retention of antioxidant compounds.

More recently, *B. catarinensis* fruits were incorporated into Brazilian mead at concentrations of 15% and 30%, followed by maturation for 30 days at 4 °C. Fruit addition modified the organic acid profile by increasing malic and citric acids and enhanced aroma through the formation or retention of fruity volatile compounds, including ethyl decanoate and ethyl dodecanoate. The formulation containing 15% *B. catarinensis* achieved the highest consumer acceptance, with an acceptance index of 86%, likely because it provided a more balanced combination of honey- and fruit-derived sensory attributes [38]. Together, these studies highlight the suitability of *Butia* spp. for the development of fermented beverages with distinctive sensory profiles and potential functional value.

### Dairy Products

Functional dairy products represent another promising application. A probiotic ice cream containing *B. odorata* pulp and *Bifidobacterium lactis* was developed, with probiotic viability remaining above recommended levels throughout 90 days of frozen storage despite the acidic pH of the product [63]. Among the formulations tested, the addition of 40% pulp provided the best balance among bioactive enrichment, structural stability, and sensory acceptance.

During storage at − 18 °C, vitamin C showed limited degradation, whereas carotenoid content increased slightly, possibly because of changes in extractability during processing or storage. In contrast, antioxidant capacity declined markedly after processing and decreased further during storage, indicating that the retention of individual bioactive compounds does not necessarily result in proportional preservation of antioxidant activity. Iron-fortified fermented dairy beverages prepared with *B. capitata* pulp also showed high acceptability among children and adolescents [69]. These findings support the feasibility of incorporating *Butia* pulp into dairy matrices while maintaining consumer acceptance and improving nutritional and functional attributes.

### Processed Food Products

Beyond fermented beverages and dairy products, *Butia* spp. pulp has been incorporated into juices, nectars, liqueurs, artisanal wines, jams, sweets, and preserves [19–21]. A study on the development of diet chewable candies containing a blend of *Butia*, blackberry, and red pitanga pulps reported high phenolic contents and satisfactory release of bioactive compounds during simulated gastrointestinal digestion, particularly during the intestinal phase [70].

These findings suggest that suitable processing conditions may partially preserve the functionality of *Butia*-derived compounds after their incorporation into processed foods. Nevertheless, information on the retention of individual bioactive compounds during commercial-scale processing remains limited.

### Natural Preservatives and Functional Ingredients

In addition to nutritional enrichment, *Butia* spp. pulp also shows potential as a source of natural preservatives and functional food ingredients. The in-situ application of an acetonic extract obtained from freeze-dried *B. odorata* pulp was evaluated on sliced mozzarella cheese inoculated with *Escherichia coli* [71]. After 72 h of refrigerated storage, treated samples showed reductions of up to 2.3 log CFU/cm^2^ compared with the control, and antimicrobial activity was maintained even when the extract was applied at the minimum inhibitory concentration. This persistence is relevant because protein- and fat-rich food matrices may reduce the effectiveness of natural antimicrobial compounds [72–75].

The antimicrobial activity was attributed primarily to the abundance of Z-10-pentadecenol and palmitic acid, compounds previously associated with bactericidal effects and inhibition of microbial growth. These findings support further investigation of *Butia*-derived extracts as potential antimicrobial ingredients for food preservation, although their sensory, toxicological, stability, regulatory, and technological performance must be established before such applications can be considered.

## Conclusions and Future Prospects

The available evidence indicates that *Butia* pulp is a source of nutrients and bioactive compounds with nutritional, technological, and functional potential. Its dietary fiber, predominantly unsaturated fatty acids, phenolic compounds, carotenoids, vitamin C, and sensory characteristics support its potential use in value-added food products. However, compositional variability among species, genotypes, maturity stages, and growing conditions, together with limited data for several species, restricts the establishment of representative profiles for the genus.

Studies have reported antioxidant activity in vitro and suggested potential anti-inflammatory, hypolipidemic, antidiabetic, chemopreventive, and antitumoral effects. However, the evidence is predominantly based on in vitro assays and a limited number of animal models, with no clinical studies confirming their physiological relevance in humans. Limited information on gastrointestinal stability, bioaccessibility, bioavailability, and metabolic fate also prevents definitive conclusions regarding these biological effects.

Freezing, modified-atmosphere storage, drying, and freeze-drying have shown potential for maintaining pulp quality and bioactive compounds, while incorporation into beverages, dairy products, confectionery, and other food matrices indicates potential for value-added applications. Nevertheless, evidence remains limited for most species and under industrial conditions.

This review is limited by its narrative design and the heterogeneity of the available studies, which restrict direct comparisons and quantitative synthesis. Future research should prioritize underexplored species, methodological standardization, industrial validation of processing and preservation strategies, and further investigation of bioaccessibility, bioavailability, clinical relevance, safety, sensory acceptance, and techno-economic feasibility. These advances could support the sustainable valorization of *Butia* pulp diversity and the development of value-added food applications. Importantly, the technological advancement of *Butia* valorization should not be restricted to large-scale industrial systems. Processing and preservation technologies should also be adapted to the technical and economic conditions of family farming, small-scale agroindustries, and local cooperatives involved in the sustainable use of native fruits. Such approaches could strengthen regional value chains, generate income for local and traditional communities, and contribute to the conservation of native ecosystems, including the Pampa and Cerrado.

## Supplementary Information

Below is the link to the electronic supplementary material.

Material Suplementar 1 (DOCX 242 KB)

Material Suplementar 2 (DOCX 48,9 KB)

## Data Availability

No datasets were generated or analysed during the current study.

## Abbreviations

ABTS: 2,2′-azinobis(3-ethylbenzothiazoline-6-sulfonic acid) assay
CF: Crude fiber
CFU: Colony-forming units
CHO: Carbohydrates
DNA: Deoxyribonucleic acid
DPPH: 2,2-diphenyl-1-picrylhydrazyl assay
DW: Dry weight
FRAP: Ferric reducing antioxidant power
FW: Fresh weight
GAE: Gallic acid equivalents
HDL: High-density lipoprotein
IL-1: Interleukin-1
INFOGEST: International standardized *in vitro* digestion method
LDL: Low-density lipoprotein
NR: Not reported
ORAC: Oxygen radical absorbance capacity
PON1: Paraoxonase 1
PRISMA: Preferred reporting items for systematic reviews and meta-analyses
SS: Soluble solids
TTA: Titratable acidity
TDF: Total dietary fiber
TSS: Total soluble solids
