# Supplementary material for: Butia spp. Pulp: Composition, Antioxidant Properties, Biological Effects, and Food Applications
Source: Plant Foods Hum Nutr. 2026 Oct 1;81(4):124. doi: 10.1007/s11130-026-01571-4 (PMC13631036; doi:10.1007/s11130-026-01571-4)
Supplement: Supplementary file 1 [file 11130_2026_1571_MOESM1_ESM.docx]

**Plant Foods for Human Nutrition**

**Review**

***Butia* spp. pulp: composition, antioxidant properties, biological effects, and food applications**

Chaiane Goulart Soares*, David Aminagat Callirgos Romero, Marjana Radünz, Elisa dos Santos Pereira, Márcia Vizzotto, Jessica Fernanda Hoffmann, Cesar Valmor Rombaldi, Chirle de Oliveira Raphaelli

*Correspondence: Chaiane Goulart Soares, Federal University of Pelotas (UFPel), R. Gomes Carneiro, No. 1, Balsa, Pelotas, 96010-610, Rio Grande do Sul, Brazil. Email: [chaianegsoares@gmail.com](mailto:chaianegsoares@gmail.com)

**Supplementary Material 1**

**Materials and Methods**

***Search strategy and study selection***

This review was conducted following the Preferred Reporting Items for Systematic Reviews and Meta-Analyses (PRISMA) 2020 guidelines as a methodological framework for the identification, selection, and reporting of relevant studies [1]. Bibliographic searches were performed in the Scopus, Web of Science Core Collection, and PubMed databases due to their broad coverage in the fields of food science and technology, nutrition, and health. The initial search was conducted on November 13, 2025, and updated in June 2026 to include newly published studies before manuscript preparation. The search strategy included studies published between 2010 and 2026. Searches were performed in the Title, Abstract, and Keywords fields (or their equivalents in each database) using the descriptor “Butia” to encompass all species belonging to this genus. The search was restricted to original research articles.

To maximize study retrieval, only the descriptor “*Butia*” was used, considering the limited number of available publications and the diversity of research topics within the genus. This broad search strategy intentionally prioritized sensitivity over specificity. Therefore, subsequent screening and eligibility assessment were specifically designed to ensure that only studies focused on *Butia* spp. pulp were included, thereby ensuring the relevance of the synthesized evidence.

Retrieved records were managed using the Rayyan web application [2] for duplicate removal and screening. Title and abstract screening were independently performed by two reviewers. Original articles focused on Butia spp. pulp addressing physicochemical composition, bioactive compounds, antioxidant activity, health-related biological effects, processing, or food applications were considered eligible. Studies unrelated to the pulp, those focused exclusively on agronomic or botanical aspects, and studies addressing other fruit fractions or by-products were excluded. Despite restricting the search to original research articles, some review papers and other non-original document types were retrieved because of database indexing and were subsequently excluded during the screening process. Figure S1 summarizes the study selection process according to the PRISMA 2020 guidelines.

Subsequently, potentially eligible studies with accessible full texts were independently assessed by both reviewers to confirm their relevance and eligibility according to the scope of this review. Disagreements were resolved by consensus through discussion and, when necessary, by reassessing the full texts. Figure S1 presents the PRISMA 2020 flow diagram of the study selection process. The included studies were qualitatively synthesized and organized into thematic categories covering physicochemical composition, bioactive compound profile, antioxidant activity, biological effects, processing, and food applications. In addition, selected review articles and other relevant publications were used as contextual support to strengthen the discussion of specific topics but were not considered part of the systematic evidence base.

Records identified from:

Databases (n = 3)

Scopus (n = 237)

Web of Science (n = 273)

PubMed (n = 90)

Registers (n = 600)

Records removed *before screening*:

Duplicate records removed (n = 263)

**Identification**

Records excluded

(n = 224)

Records screened

(n = 337)

Reports not retrieved

(n = 5)

Reports sought for retrieval

(n = 113)

**Screening**

Reports excluded (n = 51)

Studies not focused on the pulp of *Butia* (n = 40)

Studies focused exclusively on agronomic or botanical aspects (n = 11)

Reports assessed for eligibility

(n = 108)

Total studies included in review

(n = 57)

**Included**

**Figure S1*.*** PRISMA 2020 flow diagram illustrating the identification, screening, eligibility assessment, and inclusion of studies in this review.

**Reference**

1.	Page MJ, McKenzie JE, Bossuyt PM, et al (2021) Declaración PRISMA 2020: una guía actualizada para la publicación de revisiones sistemáticas. Rev Esp Cardiol 74:790–799. <https://doi.org/10.1016/j.recesp.2021.06.016>

2.	Ouzzani M, Hammady H, Fedorowicz Z, Elmagarmid A (2016) Rayyan—a web and mobile app for systematic reviews**. Syst Rev** 5:210. <https://doi.org/10.1186/S13643-016-0384-4>
