# Supplementary material for: Butia spp. Pulp: Composition, Antioxidant Properties, Biological Effects, and Food Applications
Source: Plant Foods Hum Nutr. 2026 Oct 1;81(4):124. doi: 10.1007/s11130-026-01571-4 (PMC13631036; doi:10.1007/s11130-026-01571-4)
Supplement: Supplementary file 2 [file 11130_2026_1571_MOESM2_ESM.docx]

**Plant Foods for Human Nutrition**

**Review**

***Butia* spp. pulp: composition, antioxidant properties, biological effects, and food applications**

Chaiane Goulart Soares*, David Aminagat Callirgos Romero, Marjana Radünz, Elisa dos Santos Pereira, Márcia Vizzotto, Jessica Fernanda Hoffmann, Cesar Valmor Rombaldi, Chirle de Oliveira Raphaelli

*Correspondence: Chaiane Goulart Soares, Federal University of Pelotas (UFPel), R. Gomes Carneiro, No. 1, Balsa, Pelotas, 96010-610, Rio Grande do Sul, Brazil. Email: [chaianegsoares@gmail.com](mailto:chaianegsoares@gmail.com)

**Supplementary Tables S1–S3**

**Table S1** Major fatty acids identified in *Butia* spp. pulp

| Species | Fatty acid | Relative content  (% total fatty acids) | Reference |
| --- | --- | --- | --- |
| *B. capitata* | Oleic acid (C18:1) | 41.35 | [1] |
|  | Palmitic acid (C16:0) | 23.21 |  |
|  | Linoleic acid (C18:2) | 18.33 |  |
|  | Caproic acid (C6:0) | 6.88 |  |
| *B. odorata* | Palmitic acid (C16:0) | 24.50–42.30 | [2] |
|  | Oleic acid (C18:1) | 10.40–41.00 |  |
|  | Linoleic acid (C18:2) | 10.40–24.40 |  |
|  | Linolenic acid (C18:3) | 2.20–8.30 |  |
| *B. yatay* | Oleic acid (C18:1) | 33.40 | [3] |
|  | Linoleic acid (C18:2) | 22.20 |  |
|  | Palmitic acid (C16:0) | 20.80 |  |
|  | Linolenic acid (C18:3) | 7.60 |  |

Fatty acid composition is expressed as the percentage of total fatty acids reported in each study. Range values represent the minimum and maximum concentrations observed among genotypes or independent studies

**Table S2** Bioactive compounds reported for *Butia* spp. in the selected literature

| Species | Parameter | Value | Unit | Basis | Sample | Method | Reference |
| --- | --- | --- | --- | --- | --- | --- | --- |
| *B. capitata* | Total phenolics | 493.60 ± 23.30 | mg chlorogenic acid equivalents/100 g | FW | Pulp + peel | Folin–Ciocalteu | [4] |
| *B. capitata* | Vitamin C (ascorbic acid) | 53.57 | mg/100 g | FW | Pulp + peel | HPLC-DAD | [4] |
| *B. capitata* | Total vitamin E | 121.07 | µg/100 g | FW | Pulp + peel | HPLC | [4] |
| *B. capitata* | β-Carotene | 8.56 | mg/100 g | FW | Pulp + peel | HPLC | [4] |
| *B. capitata* | Total carotenoids | 36.1 (11.1–43.9) | µg/g | FW | Pulp + thin peel | Spectrophotometry | [5] |
| *B. capitata* | β-Carotene | 16.1 | µg/g | FW | Pulp + thin peel | Spectrophotometry | [5] |
| *B. capitata* | Total phenolics (tannic acid equivalents) | 116.3 ± 37.0 (78.0–166.0) | mg/100 g | FW | Pulp (mesocarp) | Folin–Ciocalteu | [6] |
| *B. capitata* | Total phenolics (catechin equivalents) | 210.0 ± 39.0 (163.0–259.0) | mg/100 g | FW | Pulp (mesocarp) | Vanillin assay | [6] |
| *B. capitata* | Vitamin C (ascorbic acid) | 53.0 ± 18.0 (38.0–73.0) | mg/100 g | FW | Pulp (mesocarp) | 2,6-Dichlorophenolindophenol titration | [6] |
| *B. capitata* | Provitamin A | 146.2 (50.0–209.0) | RAE/100 g | FW | Pulp (mesocarp) | Carotenoid separation and spectrophotometry | [6] |
| *B. capitata* | Vitamin C (ascorbic acid) | 43 | mg/100 g | FW | Commercial frozen pulp | HPLC | [7] |
| *B. capitata* | Quercetin derivatives | 3.2 ± 0.3 | mg/100 g | FW | Commercial frozen pulp | HPLC-DAD | [7] |
| *B. capitata* | Chlorogenic acid | 0.24 ± 0.02 | mg/100 g | FW | Commercial frozen pulp | HPLC-DAD | [7] |
| *B. catarinensis* | Sum of individual phenolic compounds | 3134–4178 | mg/kg | DW | Peel + pulp, lyophilized | HPLC-DAD-MS/MS | [8] |
| *B. eriospatha* | Total phenolics | 359.50 ± 45.20 | mg gallic acid equivalents/100 g | FW | Pulp + peel | Folin–Ciocalteu | [9] |
| *B. eriospatha* | Total carotenoids | 3.85 ± 0.74 | µg β-carotene equivalents/g | FW | Pulp + peel | Spectrophotometry, 450 nm | [9] |
| *B. eriospatha* | Vitamin C (ascorbic acid) | 9.351 ± 0.06 | mg/100 g | FW | Pulp + peel | HPLC-DAD | [9] |
| *B. eriospatha* | Total phenolics | 278.4 | mg gallic acid equivalents/100 g | FW | Pulp | Folin–Ciocalteu* | [10] |

Values are presented using the original units, expression bases, analytical standards, and sample types reported by the respective studies. FW, fresh weight; DW, dry weight; RAE, retinol activity equivalents. Phenolic compounds expressed as gallic acid equivalents, chlorogenic acid equivalents, tannic acid equivalents, or catechin equivalents were not converted or pooled because these analytical expressions are method- and standard-dependent. The sum of individual phenolic compounds determined by HPLC-DAD-MS/MS was not considered equivalent to total phenolics determined by the Folin–Ciocalteu assay. Values from pulp and pulp + peel samples were kept separate, as were fresh and commercial frozen samples. No values were estimated when the respective parameter was not reported or analyzed. The value attributed to Tonietto et al. (2019) was identified through its citation in Zaccari et al. (2021); therefore, methodological details not directly confirmed from the original article are not inferred.

* Methodological information for Tonietto et al. (2019) should be interpreted cautiously because the original article was not directly available in the source set used for this reconstruction.

**Table S3** Reported antioxidant capacity of Butia spp. pulp determined by different i*n vitro* assays

| Species | Assay | Reported value | Unit | Reference |
| --- | --- | --- | --- | --- |
| *B. capitata* | ABTS | 26.00 | mM TE/g FW | [4] |
|  | DPPH | 4.74 | µmol TE/g FW | [4] |
| *B. catarinensis* | ABTS | 83.31 | µmol TE/g DW | [11] |
|  | DPPH | 14.54 | µmol TE/g DW | [12] |
|  | FRAP | 19.31 | µmol AAE/g DW | [12] |
| *B. eriospatha* | ABTS | 7.68 | mg TE/g FW | [13] |
|  | DPPH | 6.08 | mg TE/g FW | [13] |
| *B. odorata* | ABTS | 38.18-52.14 | mg TE/100 g FW | [14] |
|  | ABTS | 4.38 | mg AAE/g FW | [15] |
|  | DPPH | 160.40-610.90 | µg TE/g FW | [16] |
|  | DPPH | 785.05-3459.82 | µg TE/g FW | [17] |
|  | DPPH | 64.00 | µmol TE/g DW | [18] |
|  | DPPH | 2.58 | mg AAE/g FW | [15] |
|  | FRAP | 1.29 | mg AAE/g FW | [15] |
|  | ORAC | 265.00-278.00 | µmol TE/g DW | [18] |

Values are reported as presented in the original studies. Antioxidant activity was determined using different analytical assays and is expressed according to the units and reporting basis adopted by each study. Abbreviations: AAE, ascorbic acid equivalents; ABTS, 2,2′-azinobis(3-ethylbenzothiazoline-6-sulfonic acid) radical scavenging assay; DPPH, 2,2-diphenyl-1-picrylhydrazyl radical scavenging assay; DW, dry weight; FRAP, ferric reducing antioxidant power; FW, fresh weight; ORAC, oxygen radical absorbance capacity; TE, Trolox equivalents. Methodological note: Reported values were obtained using different extraction procedures, antioxidant assays (ABTS, DPPH, FRAP, and ORAC), calibration standards (Trolox or ascorbic acid equivalents), expression units, and reporting bases (FW or DW). Consequently, direct quantitative comparisons between studies should be interpreted with caution. The results primarily provide an overview of the antioxidant potential of *Butia* spp. pulp rather than strict interspecific comparisons

**References**

1. Lopes RM, da Silva JP, Vieira RF, et al (2012) Composição de ácidos graxos em polpa de frutas nativas do cerrado. Rev Bras Frutic 34:635–640. <https://doi.org/10.1590/s0100-29452012000200041>
2. Ferrão TS, Ferreira DF, Flores DW, et al (2013) Evaluation of composition and quality parameters of jelly palm (*Butia odorata*) fruits from different regions of Southern Brazil. Food Res Int 54:57–62. <https://doi.org/10.1016/j.foodres.2013.06.002>
3. Ouzzani M, Hammady H, Fedorowicz Z, et al (2016) Rayyan—a web and mobile app for systematic reviews. Syst Rev 5:210. <https://doi.org/10.1186/s13643-016-0384-4>
4. Barbosa MCA, Rosa QS, Cardoso LM, et al (2021) Composition proximate, bioactive compounds and antioxidant capacity of *Butia capitata*. Food Sci Technol 41:763–768. <https://doi.org/10.1590/fst.26720>
5. Faria JP, Siqueira EMA, Vieira RF, et al (2011) Fruits of *Butia capitata* (Mart.) Becc as good sources of β-carotene and provitamin A. Rev Bras Frutic 33:612–617. <https://doi.org/10.1590/s0100-29452011000500084>
6. Faria JP, Almeida F, da Silva LCR, et al (2008) Caracterização da polpa do coquinho-azedo (*Butia capitata* var capitata). Rev Bras Frutic 30:827–829. <https://doi.org/10.1590/s0100-29452008000300045>
7. Genovese MI, Da Silva Pinto M, De Souza Schmidt Gonçalves AE, et al (2008) Bioactive compounds and antioxidant capacity of exotic fruits and commercial frozen pulps from Brazil. Food Sci Technol Int 14:207–214. <https://doi.org/10.1177/1082013208092151>
8. Camboim Rockett F, de Oliveira Schmidt H, Schmidt L, et al (2020) Phenolic compounds and antioxidant activity in vitro and in vivo of *Butia* and *Opuntia* fruits. Food Res Int 137:109740. <https://doi.org/10.1016/j.foodres.2020.109740>
9. Denardin CC, Hirsch GE, da Rocha RF, et al (2015) Antioxidant capacity and bioactive compounds of four Brazilian native fruits. J Food Drug Anal 23:387–398. <https://doi.org/10.1016/j.jfda.2015.01.006>
10. Zaccari F, Del Puerto M, Cabrera MC (2021) *Butia*: physical, nutritional and antioxidant properties of red, orange and yellow fruits. Agrocienc Urug 25:e789. <https://doi.org/10.31285/agro.25.789>
11. Rockett F, Schmidt H, Rodrigues E, et al (2021) Application of refrigeration and packing can extend Butiá fruit shelf life. Food Biosci 42:101162. <https://doi.org/10.1016/j.fbio.2021.101162>
12. Danielski R, Kumari S, Sarkar S, et al (2024) Polyphenols from unconventional fruit by-products protect human epithelial intestinal cells from oxidative damage. Food Biosci 62:105302. <https://doi.org/10.1016/j.fbio.2024.105302>
13. Egea MB, Pereira-Netto AB (2019) Bioactive compound-rich, virtually unknown, edible fruits from the Atlantic Rainforest: changes in antioxidant activity and related bioactive compounds during ripening. Eur Food Res Technol 245:1081–1093. <https://doi.org/10.1007/s00217-018-3208-z>
14. Beskow GT, Hoffmann JF, Teixeira AM, et al (2015) Bioactive and yield potential of jelly palms (*Butia odorata* Barb. Rodr.). Food Chem 172:699–704. <https://doi.org/10.1016/j.foodchem.2014.09.111>
15. Ma C, Dunshea FR, Suleria HAR (2019) LC-ESI-QTOF/MS characterization of phenolic compounds in palm fruits (jelly and fishtail palm) and their potential antioxidant activities. Antioxidants 8:483. <https://doi.org/10.3390/antiox8100483>
16. Vinholes J, Lemos G, Barbieri RL, et al (2017) In vitro assessment of the antihyperglycemic and antioxidant properties of araçá, butiá and pitanga. Food Biosci 19:92–100. <https://doi.org/10.1016/j.fbio.2017.06.005>
17. Wagner JG, Cruz JG, Silveira T, et al (2022) Accessing the nutritional variability of *Butia odorata*: a food with identity. Food Sci Technol 42:e54822. <https://doi.org/10.1590/fst.54822>
18. Boeing JS, Barizão ÉO, Rotta EM, et al (2020) Phenolic compounds from *Butia odorata* (Barb. Rodr.) Noblick fruit and its antioxidant and antitumor activities. Food Anal Methods 13:61–68. <https://doi.org/10.1007/s12161-019-01515-6>
